# Linksventrikuläre Elektrodenimplantation im Rahmen der kardialen Resynchronisationstherapie

**DOI:** 10.1007/s00399-025-01088-4

**Published:** 2025-08-04

**Authors:** Andreas Napp, Henning Jansen, Till Althoff, Roland Tilz, Christian Hendrik Heeger, Victoria Johnson, Andreas Rillig, Leon Iden, Sascha Rolf, Tilman Maurer, K. R. Julian Chun, Philipp Sommer, Daniel Steven, David Duncker, Klaus K. Witte

**Affiliations:** 1 Medizinische Klinik I für Kardiologie, Angiologie und Internistische Intensivmedizin, Uniklinik RWTH Aachen, Aachen, Deutschland; 2 Elektrophysiologie Bremen, Herzzentrum Bremen am Klinikum Links der Weser, Senator-Wessling-Straße 1, 28277 Bremen, Deutschland; 3 Klinik für Kardiologie u. Angiologie, Charité – Universitätsmedizin Medizin Berlin, Berlin, Deutschland; 4 Arrhythmia Section, Cardiovascular Institute (ICCV), CLÍNIC – University Hospital Barcelona, Barcelona, Spanien; 5 Klinik für Elektrophysiologie, Medizinische Klinik II, Universitäres Herzzentrum Lübeck, Universitätsklinikum Schleswig-Holstein (UKSH), Lübeck, Deutschland; 6 Department für Rhythmologie, Abteilung für Kardiologie & Internistische Intensivmedizin, Asklepios Klinik Altona, Hamburg, Deutschland; 7 Universitäres Herz- und Gefäßzentrum, Med. Klinik III, Kardiologie und Angiologie, Universitätsmedizin Frankfurt, Frankfurt, Deutschland; 8 Universitäres Herz- und Gefäßzentrum Hamburg, Universitätsklinikum Eppendorf Hamburg, Hamburg, Deutschland; 9 Klinik für Kardiologie, Herz- und Gefäßzentrum Bad Segeberg, Bad Segeberg, Deutschland; 10 Klinik für Innere Medizin – Kardiologie, DRK Kliniken Berlin Westend, Spandauer Damm 130, 14050 Berlin, Deutschland; 11 Klinik für Kardiologie, Asklepios Klinik St. Georg, Hamburg, Deutschland; 12 Cardioangiologisches Centrum Bethanien – CCB, Frankfurt am Main, Deutschland; 13 Med. Klinik für Elektrophysiologie/Rhythmologie, Herz- und Diabeteszentrum NRW, Ruhr-Universität Bochum, Bad Oeynhausen, Deutschland; 14 Abteilung für Elektrophysiologie, Herzzentrum der Uniklinik Köln, Köln, Deutschland; 15 Hannover Herzrhythmus Centrum, Klinik für Kardiologie und Angiologie, Medizinische Hochschule Hannover, Hannover, Deutschland; 16 Leeds Institute for Cardiovascular and Metabolic Medicine, University of Leeds, LIGHT building, Clarendon Way, LS2 9JT Leeds, Großbritannien

**Keywords:** Kardiale Resynchronisationstherapie, CRT, LV-Sonde, Koronarsinus, Herzinsuffizienz (HFrEF), Cardiac resynchronization therapy, CRT, LV lead, Coronary sinus, Heart failure (HFrEF)

## Abstract

Die kardiale Resynchronisationstherapie (CRT) ist ein wichtiger Bestandteil der Therapie herzinsuffizienter Patienten mit reduzierter linksventrikulärer Ejektionsfraktion und verbreitertem Kammerkomplex bei Schenkelblock. Der Positionierung der linksventrikulären Stimulationselektrode, die in Seitästen des Koronarsinus (CS) positioniert wird, kommt hierbei eine besondere Bedeutung zu, da eine optimale Positionierung entscheidend für den Therapieerfolg ist und bei Fehlpositionierung sogar prognoseverschlechternd sein kann. Die Implantation der Elektrode in den CS ist insbesondere, wenn man die Therapie erlernt, herausfordernd und hat eine flachere Lernkurve als die Implantation eines 2‑Kammer-Geräts. Deshalb ist neben dem theoretischen Wissen auch operative Erfahrung und manuelle Geschicklichkeit wichtig. Der vorliegende Artikel soll Ihnen helfen, die Grundlagen der Implantation bis in die Tiefe zu verstehen. Sondierungstechniken auch schwieriger CS-Anatomien werden vorgestellt und die typischen Herausforderungen wie Venenklappen und typische Komplikationen an Beispielen näher beleuchtet. Zudem werden die verschiedenen Sondentypen mit deren Vor- und Nachteilen vorgestellt. Wir hoffen, Ihnen hiermit Tipps und Tricks an die Hand geben zu können, um schnell operativ erfolgreich die CRT-Therapie anwenden zu können.

## Abstract (en)

Cardiac resynchronization therapy (CRT) is an important component of the treatment of heart failure patients with reduced left ventricular (LV) ejection fraction and a widened ventricular complex due to bundle branch block. The positioning of the LV lead, which is placed in lateral branches of the coronary sinus (CS), is of particular importance, as optimal positioning is crucial for the success of therapy; poor or incorrect positioning can worsen the prognosis. CS cannulation and LV lead implantation is particularly challenging when learning the procedure, and overall, has a flatter learning curve compared to the implantation of a dual-chamber device. Therefore, in addition to theoretical knowledge, surgical experience and manual dexterity are also important. This article is intended to provide the reader a comprehensive understanding of the fundamental principles of implantation. Techniques for cannulation of challenging CS anatomies are presented, and routinely encountered difficulties such as venous valves and common complications are described using examples. Additionally, we will describe the different types of leads and their respective benefits and disadvantages. We hope to provide tips and tricks to facilitate the swift and successful application of CRT therapy in daily practice.

Die kardiale Resynchronisationstherapie (CRT) ist eine Behandlung für Patienten mit Herzinsuffizienz mit reduzierter Ejektionsfraktion (HFrEF) und inter- und intraventrikulärer elektrischer Leitungsverzögerung und basiert auf soliden Evidenzen aus randomisierten, placebokontrollierten Studien [1]. CRT, auch bekannt als biventrikuläres Pacing, wird über ein dauerhaft implantiertes kardiales implantierbares elektronisches Gerät (CIED) durchgeführt, bei dem es sich entweder um einen Herzschrittmacher oder einen Defibrillator handeln kann. Sie hat eine Klasse-Ia-Indikation für Patienten mit linksventrikulärer (LV) systolischer Dysfunktion mit einer Ejektionsfraktion (LVEF) ≤ 35 % und einem Linksschenkelblock (LBBB) mit einem QRS > 150 ms [2]. Für Patienten mit einem QRS < 150 ms, aber > 130 ms, sowie für diejenigen mit einem QRS > 150 ms, jedoch ohne klassischen LBBB, hat die CRT eine Klasse-IIa-Indikation. CRT ist nicht indiziert (und kann tatsächlich schädlich sein) bei Patienten mit einem QRS < 130 ms bei LBBB [3], oder < 150 ms bei Patienten ohne LBBB, selbst wenn eine Dyssynchronie im Echokardiogramm vorliegt. Andererseits wird allgemein akzeptiert, dass eine Resynchronisationstherapie Patienten angeboten werden sollte, die eine Schrittmacherindikation haben, eine mäßig beeinträchtigte linksventrikuläre Ejektionsfraktion (LVEF < 40 %) aufweisen und bei denen ein hoher Anteil an ventrikulärer Stimulation erwartet wird [4]. Die Prävalenz von Vorhofflimmern ist im Kollektiv der CRT-Patienten aufgrund der bestehenden strukturellen Herzerkrankung größer. Patienten mit einer schlechten Frequenzkontrolle können hier nicht oder nur eingeschränkt profitieren, da eine linksventrikuläre Nachstimulation (sog. VVT-Modus) die elektrische Dyssynchronie nicht erfolgreich aufhebt. Gleichwohl kann in Kombination mit einer Katheterablation, einer antiarrhythmischen Therapie oder im Falle eines permanenten, nicht frequenzkontrollierten Vorhofflimmerns mit einer AV-Knoten-Ablation eine sehr gute Therapieeffizienz erreicht werden.

CRT zielt darauf ab, die synchrone Kontraktion zwischen den Ventrikeln durch elektrische Stimulation wiederherzustellen und dadurch das Herzzeitvolumen sowohl in Ruhe als auch bei Aktivität zu verbessern. Zentraler Erfolgsfaktor der CRT ist die präzise Platzierung der linksventrikulären (LV) Elektrode an der lateralen Wand des linken Ventrikels durch den Koronarsinus (CS) optimalerweise am Ort der spätesten elektrischen Erregung. Durch die Verbesserung des Leitungsmusters kann CRT das Herzzeitvolumen, die Mitralinsuffizienz, die linksventrikuläre Funktion und LV-Diameter verbessern [5–7] und somit die Belastbarkeit steigern [8, 9], Symptome und Hospitalisierungen reduzieren sowie die Lebensqualität und -dauer verbessern. Die eindeutigen Mortalitätsvorteile haben die CRT von einer Behandlung für schwer beherrschbare Symptome zu einer Standardtherapie gemacht, die zusammen mit Betablockern, ACE-Hemmern, ARNI, SGLT2-Antagonisten und Aldosteronantagonisten routinemäßig für Patienten mit aktueller oder vorheriger schwerer chronischer Herzinsuffizienz eingesetzt wird. Es ist mittlerweile belegt, dass Versuche, Patienten vorab zu identifizieren, die ein „wahrscheinliches Ansprechen“ auf die Resynchronisationstherapie im Hinblick auf Symptome oder LV-Remodeling zeigen, nicht präzise sind [10]. Ein solches Vorgehen birgt das Risiko, Patienten nicht mittels CRT zu behandeln, bei denen eine Stabilisierung der Krankheit die Lebenserwartung erheblich verbessern kann [11, 12], einschließlich älterer Menschen [13].

Trotz der Einführung und Begeisterung für die Stimulation des spezifischen Reizleitungssystems („conduction system pacing“, CSP), bleibt die CRT in Ermangelung direkter Vergleichsstudien und angesichts weiterhin offener Fragen zu Erfolgsrate [14] und Haltbarkeit [15] von CSP [16] der Versorgungsstandard für Patienten, die die vorgenannten Kriterien erfüllen. Die Dauer und Erfolgsrate des Eingriffs, die Wahl der Elektrode und der Einsatz von LV-Elektroden-Fixierungen in relevanten Situationen spielen eine entscheidende Rolle für die Auswirkungen von CRT auf Bevölkerungsebene sowie für die Leistung des Geräts und die Ergebnisse bei individuellen Patienten.

In Deutschland werden pro Jahr knapp 10.000 CRT-Systeme implantiert [17], davon ca. 30 % CRT mit reiner Schrittmacherfunktion (CRT-P). Ein Drittel aller CRT-System-Implantationen sind Upgrades von bestehenden Systemen [18, 19], bei denen die Vorteile der Prozedur denen von De-novo-Implantation ähneln [20, 21]. Das additive periprozedurale Risiko einer CRT-Aufrüstung bezüglich z. B. Systeminfektionen und anderer operativer Komplikationen ist jedoch wesentlich höher [22]. Im Jahr 2019 führte die Hälfte aller implantierenden Zentren weniger als 20 Eingriffe pro Jahr durch [21]. In vorherigen EP-Basics-Ausgaben wurden bereits venöse Zugangswege, perioperatives Management sowie die Platzierung von Elektroden im rechten Ventrikel und rechten Vorhof bei aktiven Herzrhythmusimplantaten („cardiac implantable electronic devices“, CIED) thematisiert [23–25].

In diesem Artikel werden wir die Implantationstechnik und die derzeit verfügbaren Elektrodenoptionen, einschließlich solcher mit aktiven Fixierungsmechanismen, untersuchen und häufige Herausforderungen im Zusammenhang mit Zugang, Platzierung und Stabilität der Elektroden ansprechen.

## Mechanismus der CRT

**Abb. 1:**
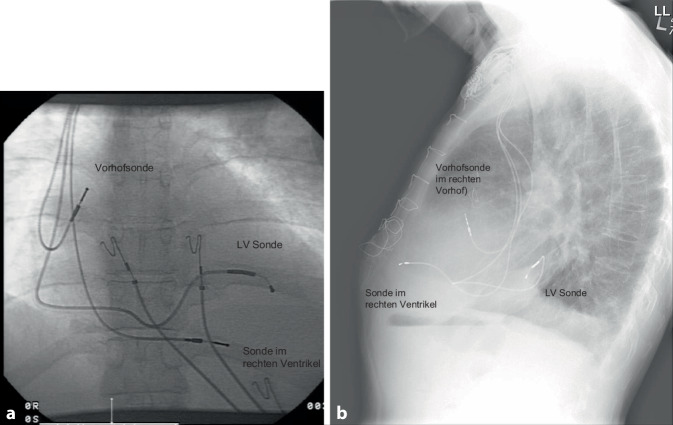
**a** Röntgenaufnahme des Thorax in p.-a., welches die 3 Elektroden eines CRT-Schrittmachers in loco typico zeigt. **b** Laterale-Thorax-Rröntgenaufnahme; die 3 Elektroden eines CRT-Schrittmachers liegen an typischer Stelle und die posteriore Lage der CS Elektrode wird deutlich

CRT verbessert die Herzfunktion, indem sie die ventrikuläre Kontraktion bei Patienten mit Herzinsuffizienz synchronisiert, insbesondere bei Patienten mit Linksschenkelblock (LBBB) oder anderen Formen elektrischer Dyssynchronie. Das CRT-Gerät umfasst typischerweise 3 Elektroden: eine im rechten Vorhof (RA), eine im rechten Ventrikel (RV) und eine im Koronarsinus zur Stimulation der lateralen Wand des linken Ventrikels (Abb. 1). Bei Patienten mit permanentem Vorhofflimmern (AF) kann auf die Vorhofelektrode verzichtet werden, und der entsprechende Anschluss am Gerät kann blind verschlossen werden.

Insgesamt ist es entscheidend, die laterale Wand des linken Ventrikels zur richtigen Zeit im Herzzyklus zu stimulieren, um die Kontraktion zu synchronisieren und das Herzzeitvolumen zu verbessern. Die klinische Wirksamkeit von CRT hängt jedoch nicht nur von der Programmierung des Geräts ab, sondern wesentlich von der korrekten Positionierung und Stabilität der LV-Elektrode im Koronarsinussystem.

## Präoperative Vorbereitung

Die Patienten sollten auch aufgrund der meist schwereren kardialen Grunderkrankung mit einem sicheren Monitoring ausgestattet werden. Es empfiehlt sich das Kleben von Defi-Patch-Elektroden und mindestens eines 3‑Kanal-EKGs. Aufgrund der elektrokardiographisch guten Abschätzung der Stimulationsposition und der Eröffnung eines möglichen Wechsels auf eine Stimulation des spezifischen Reizleitungssystems bei frustraner CS-Sonden-Implantation ist das primäre Anlegen eines 12-Kanal-EKGs sehr empfehlenswert. Unabdingbar bei Analgosedierung ist eine periphere Sauerstoffmessung über einen Finger- oder Ohrsensor. Zudem ist ein funktionierender Venenzugang (mindestens 20G, besser 18G) optimalerweise auf Implantationsseite zur Ermöglichung einer intraoperativen Phlebographie und Applikation von Medikamenten und Flüssigkeit als mandatorisch anzusehen.

## Das Implantationsverfahren

Bei einem De-novo-Patienten sollten zuerst die RV- und ggf. die RA-Elektrode implantiert werden. Dies ist vor allem deswegen zu präferieren, da bei Patienten mit Linksschenkelblock (LBBB) eine mechanische Verletzung des rechten Tawara-Schenkels während der Intubation des Koronarsinus zu einem vollständigen AV-Block führen kann, wodurch ein Routineeingriff zu einem Notfall werden kann. Für weitere Details wird auf frühere Publikationen zur Platzierung der RV- und RA-Elektroden verwiesen [23–25]. In diesem Kontext sei darauf hingewiesen, dass eine septale Sondenlage der RV-Elektrode in einer retrospektiven Analyse von 563 CRT-D-Patienten zu einer größeren Reduktion der QRS-Breite führte als eine apikale Lage, wenn sich auch beide Gruppen im Outcome nicht unterschieden [26]. Eine septale RV-Sondenlage könnte demnach förderlich sein. Prospektive randomisierte Untersuchungen hierzu fehlen bislang. Diese Elektroden können vor dem nächsten Schritt fixiert oder bis nach der LV-Implantation unfixiert bleiben.

### Intubation des Koronarsinus

**Abb. 2:**
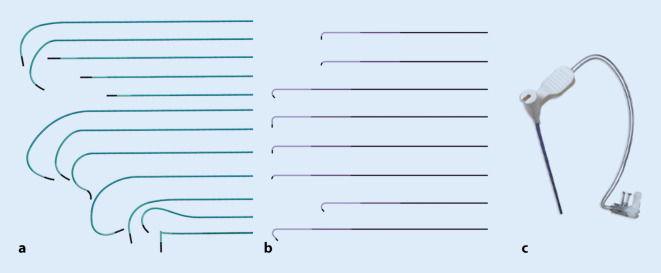
**a** CS-Guides, **b** Subselektionskatheter und **c** das Hämostaseventil. Alle Produkte sind von Medtronic

Im nächsten Schritt muss der Koronarsinus intubiert werden. Dies erfolgt mit einem speziellen Führungskatheter (Guidekatheter; Abb. 2), durch den bei Bedarf verschiedene Führungsdrähte, ein Koronarkatheter oder ein steuerbarer elektrophysiologischer (EP) Katheter eingeführt werden können, um den Koronarsinus zu lokalisieren und zu sondieren. Hierbei kann zur besseren Lokalisierung des CS-Ostiums auch Kontrastmittel über den Führungskatheter oder den Koronarkatheter appliziert werden. Diese Guides sind mit einem Hämostaseventil ausgestattet, welches die Einführung von Kathetern, Drähten und Elektroden ermöglicht, ohne dass Blut zurückfließt oder Luft eintritt. Die Wahl der Form und Länge des Führungskatheters kann anhand der Größe des rechten Atriums erfolgen. Basierend auf der erreichten Position kann bei Bedarf auf eine andere Form gewechselt werden. Alternativ kann auch nach Präferenz des Operateurs ein Ansatz verfolgt werden, bei dem stets mit einer vertrauten Katheterform begonnen wird. So wird beispielsweise von einigen Operateuren ein Multi-Purpose-Guide verwendet und das Aufsuchen des CS-Ostiums mit einem steuerbaren EP-Katheter realisiert. Andere präferieren bei linksseitiger Implantation eine steilere Kurve (z. B. „extended hook guide“; Abb. 2), da häufig eine rechtsatriale Dilatation vorliegt und mit Rotation gegen den Uhrzeigersinn und Rückzug des Katheters auch ein weniger steil abgehender CS erfolgreich intubiert werden kann. Für rechtsseitige Implantationen bieten die Hersteller spezielle Katheterformen an. Für sehr steil abgehende CS bietet sich bei kleinem RA in Einzelfällen auch von links ein MP-Rechts-Guide an.

**Abb. 3:**
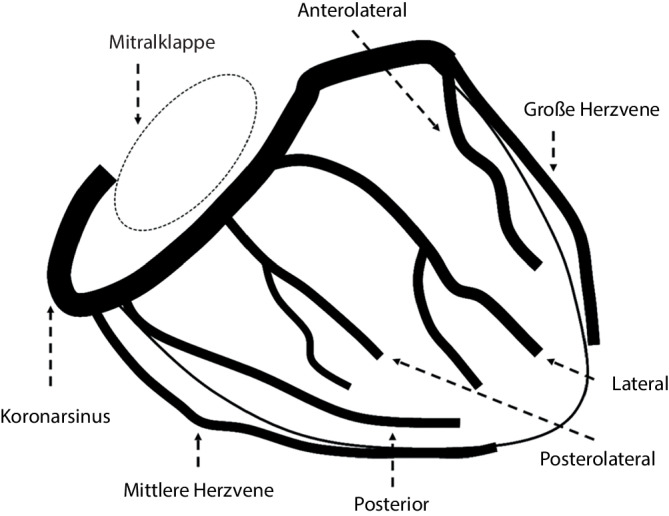
Anatomie des CS. (Aus [27] mit freundl. Genehmigung)

Als Orientierung befindet sich das CS-Ostium posterior-inferior zur Trikuspidalklappenebene. Der Koronarsinus verläuft dann inferior und posterior entlang der Trikuspidalklappenebene und weiter zur Mitralklappenebene (Abb. 3). In einer LAO-30°-Angulation der Röntgenanlage kann das CS-Ostium gut lokalisiert werden. Aus Strahlenschutzgründen und aufgrund der störenden Bildwandlerposition bei Angulation ist eine primäre Operation in p.-a.-Ausrichtung empfehlenswert. Wenn der Eingriff von der linken V. subclavia aus durchgeführt wird, werden der Katheter oder der Guide gegen den Uhrzeigersinn gedreht und vorsichtig vorgeschoben, wenn sich die Spitze in der Nähe der Trikuspidalklappe befindet. Der Einsatz eines Koronarkatheters oder eines steuerbaren EP-Katheters kann eine zusätzliche dreidimensionale Orientierung bieten und schwierige anatomische Herausforderungen insbesondere bei dilatierten Herzhöhlen lösen. Sollte das CS-Ostium nicht lokalisiert werden können, kann eine Darstellung via Femoralvenen mit einem AL-1- oder AL-2-Katheter helfen. Alternativ kann man auch eine Koronarangiographie präferenziell der linken Koronararterie in einer LAO-40°- und RAO-30°-Angulierung durchführen und dann bei langer Filmaufnahme die Kontrastierung des CS abwarten, um eine Orientierung zu erhalten. Intrakardiale (ICE) oder transösophageale Echokardiographie (TEE) können bei bildgebungserfahrenen Operateuren helfen, sind aber in der Regel nicht erforderlich und im Falle des ICE invasiv und kostenintensiv.

**Abb. 4:**
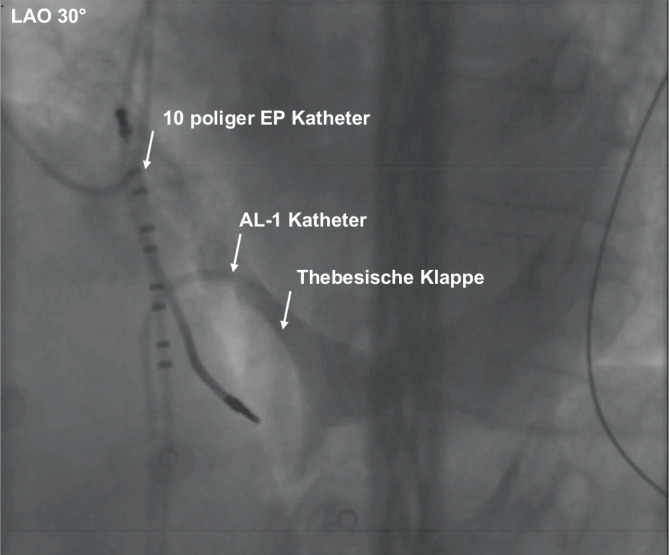
Thebesische Klappe, die über einen femoralen Zugang mittels AL-1-Katheter dargestellt wurde. Ebenfalls einliegend 10-poliger Inquiry-Katheter der Firma Abbott

Sobald das CS-Ostium lokalisiert wurde, kann eine kleine Menge Kontrastmittel dabei helfen, den Verlauf des Koronarsinus darzustellen. Das CS-Ostium ist bei einzelnen Patienten aufgrund einer ostialen Venenklappe kaum sondierbar (Thebesische Klappe). Diese Klappe ist bei den meisten Patienten klein angelegt, kann den Zugang zum CS jedoch auch subtotal verschließen. Sie kann von kranial oder von kaudal mit einem steuerbaren EP-Katheter oder einem 6‑French-Amplatz-Links-Katheter (1 oder 2) zur Seite gedrängt und dann nach Darstellung mit dem CS-Guide passiert werden (Abb. 4). Der Guide wird vorsichtig bis zur mittleren lateralen Region vorgeschoben, wobei darauf geachtet wird, Seitäste oder die atriale Vene des linken Atriums (Marshall-Vene) zu vermeiden. An diesem Punkt kann die Drehung des Katheters oder die Verwendung eines hydrophilen Drahtes innerhalb des Katheters den Vorschub desselben erleichtern. Insbesondere im proximalen Drittel ist der CS anatomisch bedingt vulnerabel und dissektions- bzw. perforationsgefährdet.

**Abb. 5:**
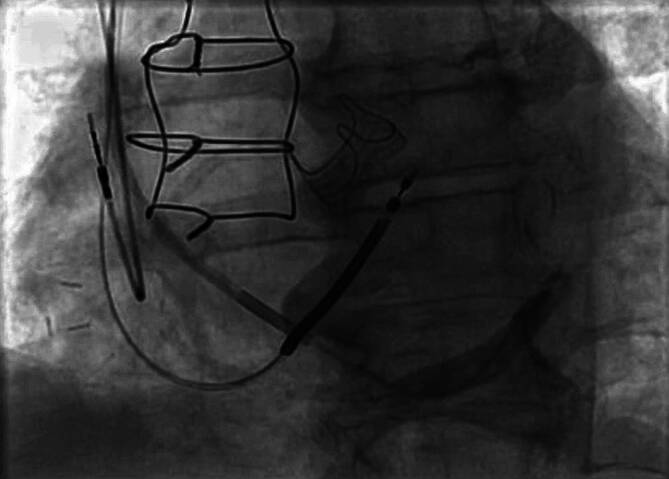
CS-Angiographie mit Darstellung der Vieussen-Klappe

Falls das Vorankommen des Guides auf mittlerer Venenhöhe schwierig ist, sollte dieser leicht zurückgezogen werden, und eine geringe Menge Kontrastmittel kann helfen, das Vorhandensein von weiteren Venenklappen an dieser Stelle zu erkennen. Die Vieussen-Klappe, die nach Abgang der Marschallvene lokalisiert ist, kann hier einen Abbruch des CS vortäuschen (Abb. 5). Diese Klappen können in der Regel mit einem hydrophilen Draht überwunden werden, der vom Katheter gestützt wird und durch die Klappe gedreht werden kann. In Einzelfällen können auch über einen Koronarkatheter steifere Drähte (Amplatz Extrastiff o. Ä.) vorgebracht werden, oder ein kräftigerer Innenkatheter verwendet werden, der den Backup des Guides beim Vorschub verbessert.

### Ballonokklusionsvenographie

**Abb. 6:**
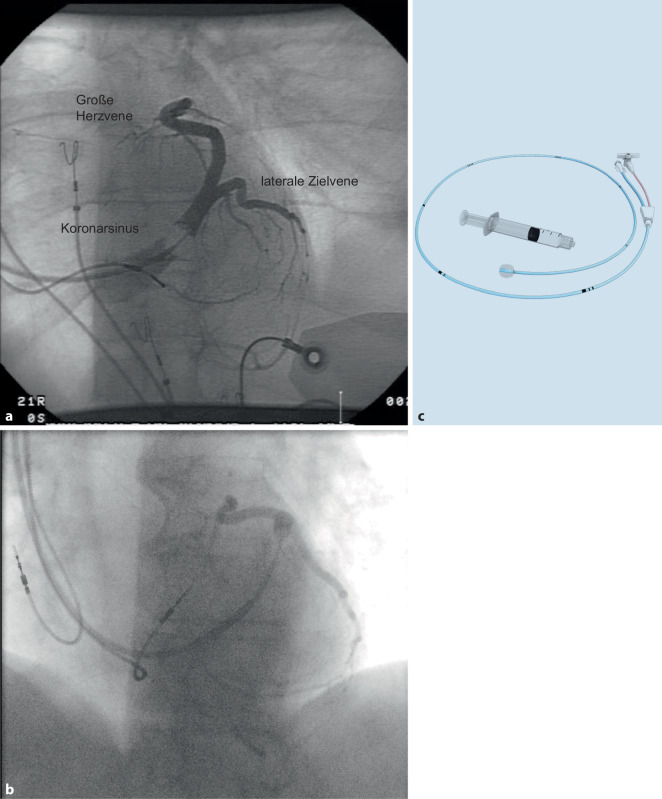
Ballonvenographie zeigt die Anatomie des Koronarsinus und eine geeignete laterale Zielvene. **a**, **b** radiologische Aufnahmen; **c** Ballon-Okklusionskatheter (Abbott)

Sobald der Guide mittig in der Vene positioniert ist, kann eine kleine Menge Kontrastmittel verwendet werden, um zu zeigen, ob er koaxial zur Vene ist. Ein Ballonokklusionskatheter wird dann eingesetzt, um eine retrograde Venographie zu ermöglichen. Dieser sollte bis zur Spitze des Guides vorgeschoben werden. Dann wird der Guide leicht zurückgezogen, um den Ballon gefahrlos zu enthüllen. Der Ballon sollte nicht routinemäßig über die Spitze hinaus vorgeschoben werden, um das Risiko einer Gefäßverletzung zu vermeiden. Ein weiterer kleiner Kontrastmittelbolus kann klären, dass sich der Ballonkatheter nicht in einer kleinen Vene befindet. Eine Kontrastmittelinjektion von etwa 10 ml reicht in der Regel aus, um die venöse Anatomie umfassend darzustellen (Abb. 6a). Das Ablassen des Ballons bei maximaler Füllung unter Fortsetzung der Röntgenaufnahme ermöglicht es, eventuell durch den Ballon okkludierte Seitenäste nicht zu übersehen. Wenn der Durchmesser des CS zu groß ist und der Ballon nicht zu einer kompletten Okklusion führt, kann auch im dünneren distalen Bereich der „great cardiac vein“ vorsichtig okkludiert werden und mit einer langen Szene über eine retrograde Füllung über Anastomosen die proximalen Venen visualisiert werden (Abb. 6b).

### Auswahl der Zielvene

Der erfolgreiche Einsatz hängt nicht nur davon ab, Zugang zum Koronarsinus zu erhalten, sondern auch davon, in die Äste mit ausreichender Größe zu navigieren, um die Elektrode mit allen Elektrodenpolen unterzubringen.

Zu den wichtigsten Herausforderungen bei der LV-Elektrodenplatzierung gehören:

- Venen-Tortuositität: Die Koronarvenen weisen oft einen gewundenen Verlauf auf, was die Komplexität der Elektrodenplatzierung erhöht und anspruchsvolle Hilfsmittel für die Navigation erfordert.
- Elektrodenstabilität: Es ist entscheidend, dass die LV-Elektrode über die Zeit stabil bleibt, um eine effektive Stimulation zu gewährleisten. Instabile Elektroden können zu schlechten Messwerten oder zu einer Dislokation der Elektrode führen, was den Erfolg der CRT beeinträchtigt.
- Anatomische Variabilität: Die Lage und Größe der Koronarvenen sind patientenindividuell, dies kann die ideale Platzierung erschweren.
- Phrenikusstimulation: Aufgrund des Verlaufes des N. phrenicus kann bei bestimmten Elektrodenpositionen eine begleitende Stimulation dieses Nervs und damit eine stimulationssynchrone Kontraktion des Zwerchfells auftreten (sog. „Zwerchfellzucken“). Posteriore, laterale und weiter apikale Elektrodenpositionen bringen hierfür ein erhöhtes Risiko mit sich. Schwierig ist, dass in aufrechter Position des Patienten und bei Inspiration eine Lageveränderung der Elektrode auftreten kann und dies während der Operation nicht detektiert wird. Aufgrund der quadripolaren Elektroden mit multiplen Stimulationsvektoren und der Möglichkeit einer Impulsdauerverbreiterung mit der dann niedrigeren erforderlichen Impulsamplitude kann dieses Problem meist ohne Revisionsoperation beherrscht werden.

Die Entscheidung, welche Vene verwendet werden soll, wird daher durch eine Kombination anatomischer Gegebenheiten und elektrischer Messwerte bestimmt.

### Entscheidung der Wahl der Elektrode

Für die CRT-Implantation stehen mehrere Elektrodenoptionen zur Verfügung, die unterschiedliche Fixierungsmechanismen aufweisen, um die Stabilität der Elektrode nach der Positionierung zu gewährleisten. Diese lassen sich grob in passive Fixierung und aktive Fixierung unterteilen, wobei beide Vor- und Nachteile haben.

#### Passive Fixierungselektroden

**Abb. 7:**
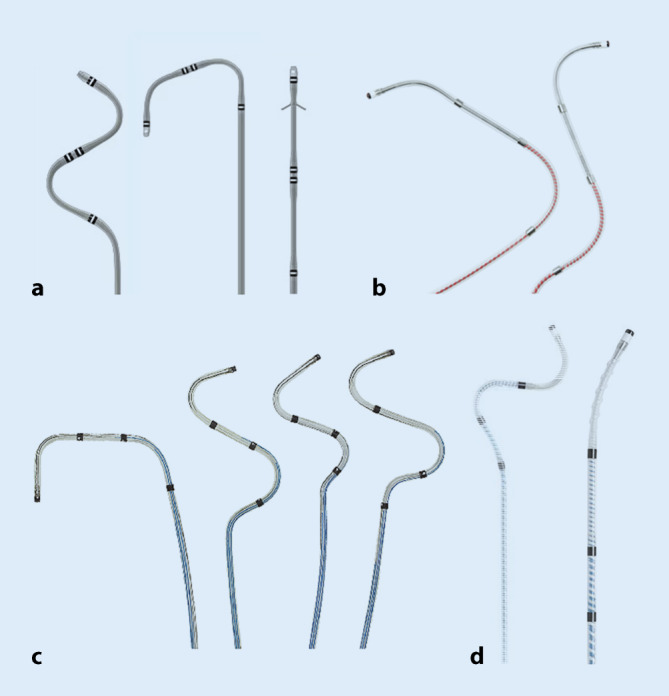
Linksventriculaere(LV)-Sonden mit unterschiedlichen Kurvenformen. **a** Medtronic, **b** Biotronik, **c** Abbott, **d** Microport

Diese Elektroden sind mit flexiblen Widerhaken oder speziellen Krümmungen ausgestattet, die mit der venösen Wand in Kontakt treten, um die Elektrode an ihrem Platz zu halten. Passive Fixierungselektroden nutzen die natürliche Krümmung der Vene, um die Elektrode im Sinne einer *Verklemmung* zu stabilisieren (Abb. 7). Sie sind im Allgemeinen einfacher zu implantieren und erfordern weniger Drehmoment, um die Elektrode zu positionieren.

Vorteile:

- Einfacher zu implantieren und während der Platzierung neu zu positionieren.
- Ideal für Venen mit guter natürlicher Geometrie.
- Einfacher zu extrahieren.

Nachteile:

- Weniger stabil als aktive Fixierung, insbesondere in gewundenen oder kleinen Venen.
- Können mit der Zeit zur Dislokation neigen.

#### Aktive Fixierungselektroden

**Abb. 8:**
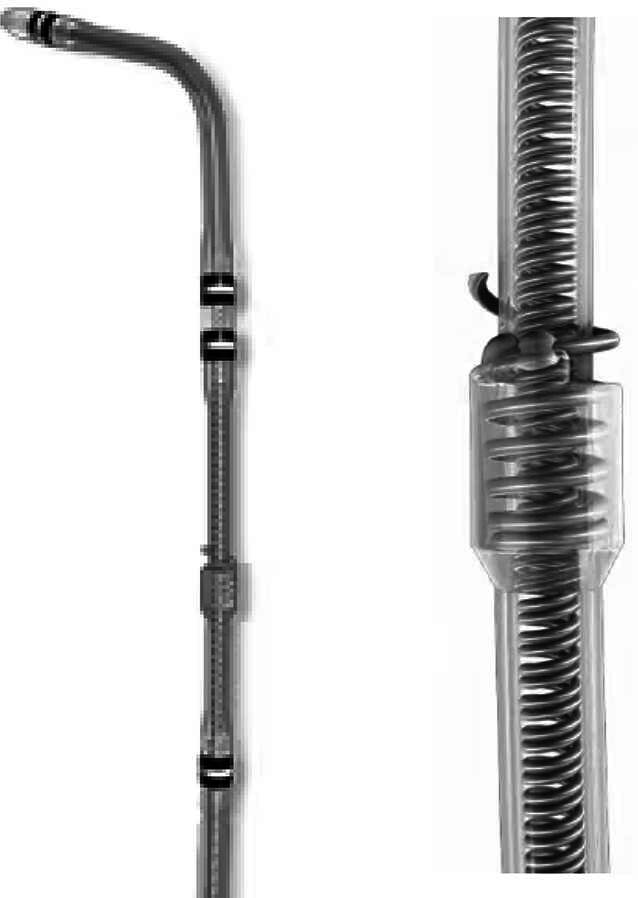
Medtronic Attain Stability quadripolare LV-Elektrode zur aktiven Fixierung im Koronarsinusast

Aktive Fixierungselektroden sind so konstruiert, dass die Elektrode mit einer kleinen helikalen Schraube, die zwischen den Elektrodenpolen liegt, gesichert wird, in dem die Elektrode rotiert wird bis die Schraube sich in das Endothel innerhalb der Vene verankert (Abb. 8). Eine aktive Fixierung bietet eine größere Stabilität im Vergleich zur passiven Fixierung und ist besonders nützlich bei Venen mit größerem Durchmesser oder kürzeren Venen mit herausfordernder Anatomie.

Vorteile:

- Erhöhte Stabilität und geringeres Risiko einer Dislokation.
- Ideal für Venen mit gewundenem oder nicht idealem Verlauf.
- Sichert die Elektrodenplatzierung selbst in anspruchsvolleren Ästen des Koronarsinus.

Nachteile:

- Potenzielles Risiko einer Gefäßverletzung, wenn nicht korrekt positioniert.
- Kann schwieriger neu positioniert oder angepasst werden, nachdem sie platziert wurde.
- Extraktionen können im langfristigen Verlauf mit einem erhöhten Risiko einhergehen.

Quadripolare Elektroden sind mittlerweile der Standard, da sie mehr Optionen hinsichtlich der potenziellen Stimulationsvektoren bieten und dadurch eine ungewollte Stimulation des N. phrenicus in der Regel vermieden werden kann. Die größere Fläche, über die diese Pole verteilt sind, ermöglicht es auch, die Elektroden tiefer in eine Vene zu platzieren, sodass der distale Pol sogar in der Nähe des Apex liegt, da die proximaleren Pole seitlich mittig positioniert sind. Insgesamt haben quadripolare Elektroden trotz ihres komplexeren Designs eine ähnliche Implantationserfolgsrate und Langlebigkeit wie ältere bipolare Elektroden. Sie zeichnen sich durch langfristig stabile elektrische Messwerte aus sowie niedrige Raten von unerwünschten Ereignissen im Vergleich zu bipolaren Elektroden [28, 29].

## Auswirkungen der Elektrodenplatzierung auf die CRT-Ergebnisse

Das Hauptziel des Verfahrens ist es, eine Elektrode auf der lateralen Seite des linken Ventrikels in einer stabilen Position zu platzieren, um eine Stimulation des N. phrenicus zu vermeiden. Die optimale Positionierung der LV-Elektrode hat einen erheblichen Einfluss auf die Wirksamkeit der CRT. Die Platzierung der Elektrode in den lateralen oder posterolateralen Bereichen des linken Ventrikels, mittig zwischen Apex und Basis, führt zu den besten Ergebnissen hinsichtlich einer verbesserten linksventrikulären Auswurffraktion (LVEF), reduzierten Krankenhausaufenthalten und besserer Langzeitüberlebensrate [30]. Allerdings, obwohl man diesen idealen Ort häufig identifizieren kann, muss dies mit dem Pragmatismus der anatomischen Erreichbarkeit, Elektrodenstabilität und Vermeidung einer N.-phrenicus-Stimulation in Einklang gebracht werden. Aufgrund der Vielzahl potenzieller Vektoren hat die Einführung quadripolarer Elektroden die Chance erhöht, eine erfolgreiche Elektrodenplatzierung zu erreichen, jedoch ist in einigen Fällen ein Kompromiss erforderlich. Ein höhere Stimulationsreizschwelle und eine nur suboptimale Platzierung verwendeten Elektrodenpole sind akzeptabel, wenn eine gute Elektrodenstabilität gewährleistet ist und keine oder nur eine beherrschbare N.-phrenicus-Stimulation auftritt. Umgekehrt kann eine suboptimale Elektrodenplatzierung, insbesondere in apikalen Segmenten oder septal zu einer weniger effektiven Resynchronisation und einem geringeren klinischen Nutzen führen [31], obwohl die Studienlage in diesem Bereich uneinheitlich ist [32].

Während einige Studien gezeigt haben, dass eine gezielte Elektrodenplatzierung unter Verwendung von echokardiographischem Speckle-Tracking [33] oder Magnetresonanztomographie [34, 35] die klinische oder echokardiographische Ergebnisse verbessern kann, haben andere keinen Nutzen gezeigt, trotz der zusätzlichen Komplexität des Versorgungswegs, die mit einem multimodalen Ansatz verbunden ist [36, 37]. Deshalb, während Ergebnisse laufender Studien noch ausstehen [38], ist ein gezielter Ansatz für die Elektrodenposition nicht routinemäßig, nicht zuletzt, weil die ideale Position häufig nicht erreicht werden kann oder mit einer anderen Herausforderung sowie die Phrenikusstimulation verbunden ist. Daher ist es angesichts der begrenzten Evidenz, dass eine gezielte Elektrodenplatzierung die Ergebnisse verbessert, nicht üblich, eine optimierte anatomische Sondenposition im Voraus zu planen, da auch die Stabilität der Elektrode und die Vermeidung der Phrenikusstimulation mitentscheidend sind.

## Elektrodenplatzierung

Sobald eine stabile Position des Guides im Koronarsinus erreicht wurde und die Anatomie klar ist, wird die gewählte Elektrode platziert. Die Platzierung der Elektrode im Zielgefäß wird durch die Verwendung eines Angioplastiedrahts mit einer leicht gebogenen Spitze (oder einer vorgefertigten J‑Form) erleichtert, um das Steuern in die lateralen Äste zu ermöglichen. Der Draht wird in die Zielvene vorgeschoben und die Elektrode dann über diesen Draht bis zur Stabilität oder der gewünschten Position (vorzugsweise beides) vorgebracht.

**Abb. 9:**
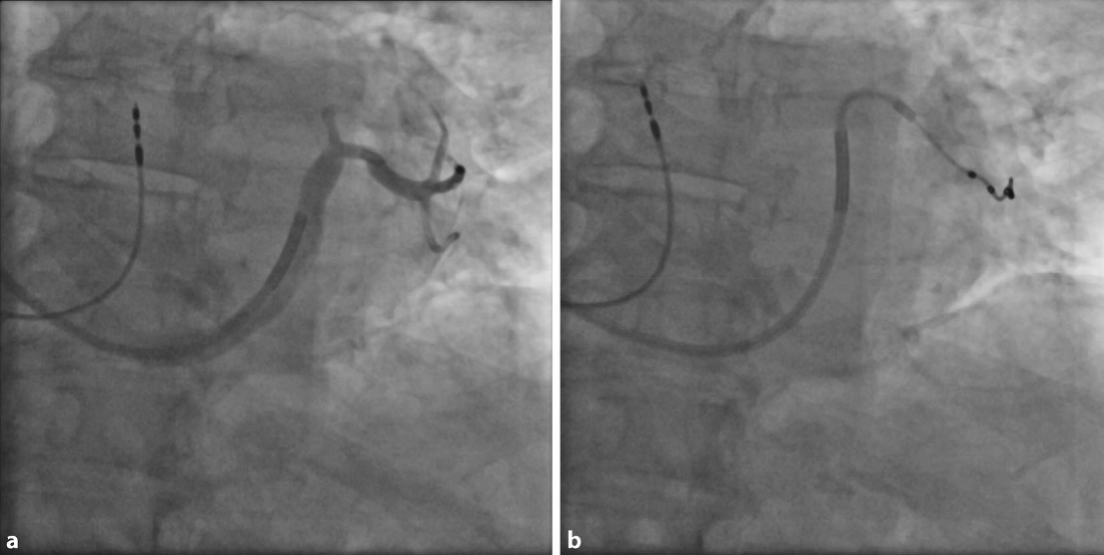
**a** Darstellung der lateralen Zielvene über einen 130° Subselektionskatheter. **b** Platzierte CS-Elektrode über den Subselektionskatheter. Dieser gibt einen guten Backup für das Vorbringen der Elektrode

Elektroden in lateralen Venen mit einem spitzwinkligen Abgang zum Koronarsinus können besonders schwierig zu platzieren sein, da der Vorwärtsdruck auf die Elektrode dazu führen kann, dass sie in den Hauptanteil des Koronarsinus prolabiert. Um dies zu vermeiden, gibt es mittlerweile eine Vielzahl von Subselektionskathetern mit einem ausreichend großen Innendurchmesser, um das Einführen der Elektrode zu ermöglichen. Diese bieten ausreichend Unterstützung an der *oberen* Krümmung, um die Kanülierung dieser schwierigen Venen zu erleichtern (Abb. 9a). Die Wahl des Führungsdrahtes, über den die Elektrode geführt wird, kann ebenfalls den Implantationserfolg beeinflussen. Weiche Drähte ermöglichen in der Regel die leichte Sondierung auch kleiner Gefäße mit schwierigen Abgängen. Gewundene Venen können gut mit einem *Extra-support-Draht* teilweise begradigt werden und bieten gleichzeitig ein gutes Backup für den Elektrodenvorschub. Steifere Führungsdrähte sind oft nicht so gut steuerbar. Sobald die Elektrode positioniert ist, wird unter Durchleuchtung der Führungsdraht zurückgezogen (Abb. 9b).

Falls eine Elektrode mit aktiver Fixierung gewählt wurde, muss der Mechanismus durch Drehen der gesamten Elektrode entweder mit dem Führungsdraht oder der bereitgestellten Stilette aktiviert werden. Um zu bestätigen, dass diese fixiert ist, führt ein sanftes Ziehen dazu, dass der Guide sich in Richtung der Spitze der Elektrode bewegt, und ein sanftes Drücken sollte dazu führen, dass die Spitze des Guides in Richtung des Koronarsinusostiums verschoben wird.

## Abschluss der Elektrodenplatzierung

**Abb. 10:**
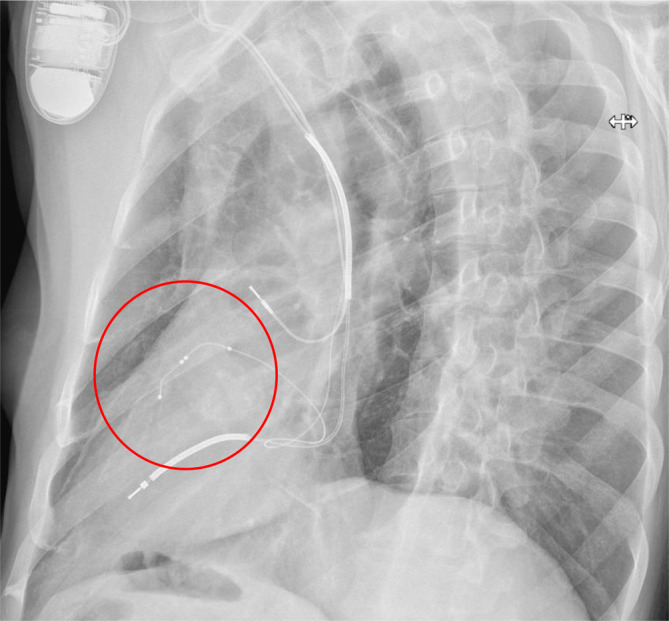
Laterale Röntgenaufnahme des Thorax, die eine unbefriedigende anteriore Position der LV-Elektrode zeigt (*roter Kreis*). Diese Position ist insbesondere mit einem schlechten Ergebnis der CRT verbunden

Sobald die Position in den LAO- und RAO-Bildern verifiziert wurde und sich auf der lateralen Wand (und nicht anterior; Abb. 10) befindet, sollten die elektrischen Messwerte erhoben werden und ein Schwellenwert für die Phrenikusstimulation bestimmt werden. Während die Stimulationsreizschwelle einer transvenösen epikardialen LV-Elektrode häufig höher ist als bei endokardialen Elektroden, kann diese im Allgemeinen akzeptiert werden, solange sie einen ausreichenden Abstand zur Phrenikusschwelle hat und die Elektrodenstabilität am gewünschten Ort akzeptabel ist. Darüber hinaus ist die übliche Sicherheitsmarge bei der Programmierung des LV-Outputs im Gegensatz zu RV-Elektroden von dem 2‑fachen des Reizschwellenwertes nicht unbedingt erforderlich, sodass die Programmierung darauf abgestimmt werden sollte, ein zuverlässiges LV-Capture zu erreichen. Die Impulsamplitude sollte jedoch aus Gründen der Energieersparnis unterhalb der Batteriespannung liegen. In bestimmten Fällen können höhere Reizschwellen als Kompromiss für die Elektrodenplatzierung und -stabilität akzeptabel sein.

**Abb. 11:**
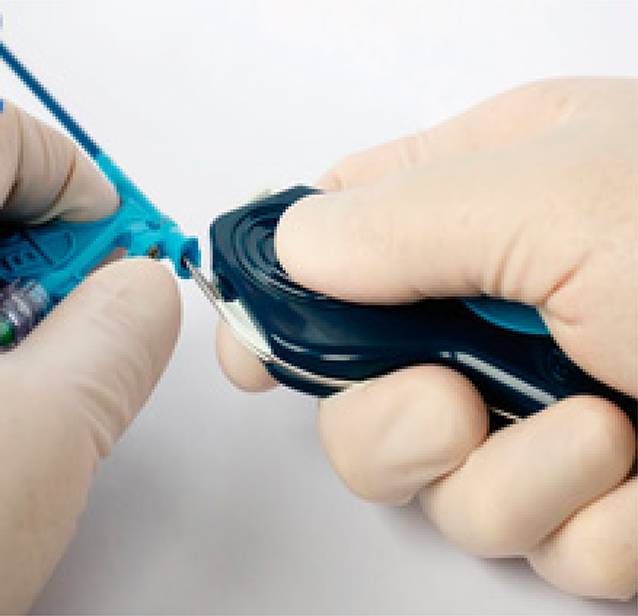
Das Schlitzmesser wird durch das Ventil vorgeschoben, und anschließend wird der Guide gleichmäßig und stetig zurückgezogen

Anschließend muss der Guide mit einem speziell angefertigten Schlitzmesser der Länge nach eröffnet werden, um die Elektrode freizulegen. Die Elektrode wird hierbei mit dem Messer in einer stabilen Position gehalten und der Guide in einer kontinuierlichen ggf. auch fluoroskopisch kontrollierten Bewegung zurückgezogen und hierbei längs aufgeschlitzt. (Abb. 11). Der Draht oder Stilette können dann entfernt und die Elektrode wie üblich an der Faszie angenäht werden. Insgesamt muss auf eine feste Fixierung der Elektroden mit einer doppelten oder besser dreifachen Annaht des Sleeves an die Muskelfaszie geachtet werden, um ein Herausarbeiten bei Armbewegungen zu verhindern.

**Abb. 12:**
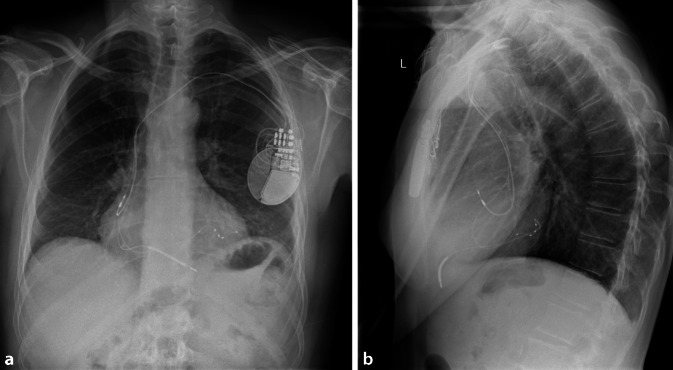
Posterior-anteriore (**a**) und laterale (**b**) Röntgenaufnahme des Thorax, die eine gute Position der LV-Elektrode bei einer De-novo-CRT-D-Implantation zeigen

**Abb. 13:**
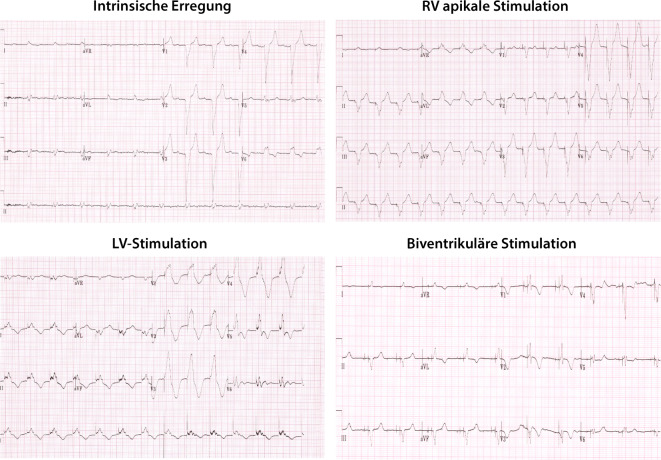
EKGs, die die intrinsische Erregung, RV-Stimulation, LV-Stimulation und biventrikuläre Stimulation zeigen

Postoperativ sollte eine PA- und laterale Röntgenaufnahme des Thorax gemacht werden, um die angemessene und stabile Position der LV-Elektrode zu bestätigen (Abb. 12). Außerdem sollte im Rahmen der ersten Nachsorge ein EKG aufgezeichnet werden, das die intrinsische Erregung, RV-Stimulation, LV-Stimulation und biventrikuläre Stimulation dokumentiert. Dies kann als Referenz z. B. für einen Stimulationsverlust der LV-Sonde herangezogen werden und zusätzlich Aufschluss über die Kammerkomplexbreite unter biventrikulärer Stimulation geben. Die RV/LV-Synchronität könnte hier optimiert werden, obwohl der klinische Nutzen im Vergleich zur AV-Zeit-Optimierung weiterhin fraglich ist (Abb. 13).

## Komplikationen

Die Operationsdauer und die Erfolgsraten stehen in engem Zusammenhang mit der Erfahrung des Operateurs. Aufgrund der aktuellen, politisch vorgegebenen Umstrukturierung in der Krankenversorgung mit Wegfall von Leistungsgruppen in einzelnen Krankenhäusern wird es zu einer Zentralisierung dieser Eingriffe kommen. Die flachen Lernkurve für solche Eingriffe erfordert zudem eine ausreichend große Fallzahl pro Operateur, um eine hochqualitative Patientenversorgung zu gewährleisten. Die Dosis-Flächen-Produkt-Grenzen für die Implantation eines De-novo-CRT-Systems sollten konstant unter 5000 cGy/cm^2^ liegen [16]. Der Operateur sollte eine sorgfältige Beurteilung des Risikos einer Infektion und der hohen Strahlendosis einer prolongierten Prozedur im Vergleich zu einem Zweiteingriff durch einen erfahreneren Operateur vornehmen [39]. Neuimplantationen und Revisionsoperation unterliegen in Deutschland einer bundeseinheitlichen externen Qualitätssicherung.

Eine frustrane LV-Elektroden-Implantation sollte in weniger als 5 % der Fälle auftreten. Die Beliebtheit des CSP nimmt zu und die Verfügbarkeit dieser Option hat scheinbar zu einer falsch erhöhten *Fehlerrate* der LV-Sonden-Platzierung geführt. Insgesamt empfehlen wir, dass im Falle eines Scheiterns der LV-Elektroden-Implantation der Fall mit einem sehr erfahrenen Operateur besprochen wird, bevor ein zweiter Versuch unternommen wird, solange CSP nicht in Bezug auf patientenorientierte Ergebnisse als gleichwertig mit CRT nachgewiesen ist. CSP stellt jedoch eine attraktive Alternative dar, um eine chirurgische epikardiale Sondenanlage, die deutlich invasiver ist, zu vermeiden.

Komplikationen, die speziell mit der Sondierung des Koronarsinus und der LV-Elektroden-Platzierung verbunden sind, beinhalten Verletzungen des Koronarsinus mit einer Dissektion oder gar Perforation. Während Letzteres sehr selten zu schwerwiegenden Ereignissen führt, kann eine signifikante Dissektion die Implantation bei fehlender Sondierbarkeit der Zielvene unmöglich machen. Ein zweiter Versuch kann nach 6 Wochen unternommen werden, wenn die Dissektion in der Regel abgeheilt ist. Drahtungsversuche bei bestehender größerer Dissektion sollten nur von erfahrenen Operateuren in der gleichen Prozedur erfolgen.

Relevante Elektrodendislokationen ggf. mit Reizschwellenproblemen und hartnäckiger N.-phrenicus-Stimulation, die zu einer Reoperation führen, sollten insgesamt unter 5 % liegen. Der Einsatz quadripolarer LV-Elektroden hat zu einer Verringerung der Reoperationen aufgrund dieser Indikationen geführt. Ein Prolaps der LV-Elektrode in den Koronarsinus oder das rechte Atrium tritt in weniger als 1 % der Fälle auf, erfordert jedoch eine Wiederholung des Verfahrens aufgrund des Risikos induzierter Arrhythmien.

## Jüngste Fortschritte und zukünftige Entwicklungen in der Resynchronisationstherapie

Das Feld der CRT hat sich mit Innovationen in der Programmierung weiterentwickelt, die die Batterielebensdauer, z. B. durch automatische Reizschwellentests mit Anpassung der Amplitude (sog. Capture-Management) und die CRT-Effektivität verbessern. Verschiedene Algorithmen zur Optimierung der AV- und VV-Zeiten werden von den Herstellern angeboten. Auch wenn diese nur mäßige Vorteile zu haben scheinen, sind sie besser als das Festhalten an der Out-of-the-box-Programmierung, die leider immer wieder beibehalten wird. Eine echokardiographisch und elektrokardiographisch gestützte Optimierung der AV- und ggf. VV-Zeit sowie Auswahl der LV-Stimulationskonfiguration sollten möglichst routinemäßig, aber insbesondere bei Nichtansprechen oder Verschlechterung durchgeführt werden [11]. Quadripolare LV-Elektroden mit aktiver Fixierung, die die Auswahl des Implantationsorts mit weniger Kompromissen ermöglichen, verbessern die Erfolgsraten. Neuere Kathetertechnologien und fortschrittliche Bildgebungstechniken, einschließlich intrakardialer Echokardiographie und MRT-kompatibler Elektroden, bieten eine höhere Präzision bei der Elektrodenplatzierung und -verfolgung während des Verfahrens. Jedoch nicht jede Innovation, die vielversprechend schien, hat sich in größeren prospektiven unabhängigen Studien als vorteilhaft gezeigt. Das Multi-Point-Pacing, das *bessere* Resynchronisation versprach, verbessert beispielsweise die klinischen Ergebnisse nicht, hat jedoch beträchtliche Kosten in Bezug auf die Batterielebensdauer und sollte nur bei Patienten aktiviert werden, die trotz optimaler Therapie eine Verschlechterung zeigen [40].

Zusätzlich könnte eine personalisierte Programmierung basierend auf patientenspezifischen anatomischen und kontraktilen Eigenschaften einen noch größeren therapeutischen Nutzen bieten, während zunehmend erkannt wird, dass die Gerätetherapie für Herzinsuffizienz am besten von einem interdisziplinären Team in einer spezialisierten Einrichtung durchgeführt wird [41].

## Fazit

Der Erfolg der kardialen Resynchronisationstherapie ist eng mit der genauen und stabilen Positionierung der linksventrikulären Elektrode verbunden. Während Elektroden mit passiver Fixierung nach wie vor ein fester Bestandteil der CRT sind, haben sich Elektroden mit aktiver Fixierung als überlegen in Bezug auf die Stabilität bei schwierigen anatomischen Bedingungen erwiesen. Diese Technologien bringen jedoch ihre eigenen Herausforderungen mit sich, insbesondere bei verwundenen oder kleinen Koronargefäßen, und neben den verwendeten Materialien ist die Erfahrung und Geschicklichkeit des Operateurs maßgeblich.
